# Exploring the Regulatory Dynamics of *BrFLC*-Associated lncRNA in Modulating the Flowering Response of Chinese Cabbage

**DOI:** 10.3390/ijms25031924

**Published:** 2024-02-05

**Authors:** Yun Dai, Xinyu Gao, Shifan Zhang, Fei Li, Hui Zhang, Guoliang Li, Rifei Sun, Shujiang Zhang, Xilin Hou

**Affiliations:** 1 National Key Laboratory of Crop Genetics & Germplasm Innovation and Utilization, Key Laboratory of Biology and Genetic Improvement of Horticultural Crops (East China), Ministry of Agriculture and Rural Affairs of China, Engineering Research Center of Germplasm Enhancement and Utilization of Horticultural Crops, Ministry of Education of China, Nanjing Agricultural University, Nanjing 210095, China; daiyun1210@126.com; 2 State Key Laboratory of Vegetable Biobreeding, Institute of Vegetables and Flowers, Chinese Academy of Agricultural Sciences, Beijing 100081, China; gaoxy7078@163.com (X.G.); zhangshifan@caas.cn (S.Z.); lifei@caas.cn (F.L.); zhanghui05@caas.cn (H.Z.); liguoliang@caas.cn (G.L.); sunrifei@caas.cn (R.S.)

**Keywords:** vernalization, lncRNA, Chinese cabbage, *LncFLC*, *BrFLC*

## Abstract

Vernalization plays a crucial role in the flowering and yield of Chinese cabbage, a process intricately influenced by long non-coding RNAs (lncRNAs). Our research focused on *lncFLC1*, *lncFLC2a*, and *lncFLC2b*, which emerged as key players in this process. These lncRNAs exhibited an inverse expression pattern to the flowering repressor genes *FLOWERING LOCUS C 1* (*BrFLC1*) and *FLOWERING LOCUS C 2* (*BrFLC2*) during vernalization, suggesting a complex regulatory mechanism. Notably, their expression in the shoot apex and leaves was confirmed through in fluorescent in situ hybridization (FISH). Furthermore, when these lncRNAs were overexpressed in *Arabidopsis*, a noticeable acceleration in flowering was observed, unveiling functional similarities to *Arabidopsis*’s *COLD ASSISTED INTRONIC NONCODING RNA* (*COOLAIR*). This resemblance suggests a potentially conserved regulatory mechanism across species. This study not only enhances our understanding of lncRNAs in flowering regulation, but also opens up new possibilities for their application in agricultural practices.

## 1. Introduction

In plant biology, the intricate regulation of gene expression is fundamental to understanding plant development, phenotypic plasticity, and adaptation to environmental stressors. Among the various molecular players, long non-coding RNAs (lncRNAs) have garnered significant attention [1,2,3]. These RNA molecules, typically longer than 200 nucleotides and lacking significant protein-coding capacity, have been implicated in a myriad of regulatory functions [4,5]. They modulate gene expression through diverse mechanisms, including chromatin remodeling, transcriptional interference, and post-transcriptional modifications, thereby influencing key developmental and physiological pathways [6].

The flowering process, a pivotal developmental transition in angiosperms, is orchestrated by an intricate network of genetic and epigenetic mechanisms. Within this complex, lncRNAs have risen to prominence as key regulatory elements, influencing various facets of plant development, particularly the timing and nature of flowering [7,8,9]. The multifaceted role of lncRNAs in modulating flowering time, determining floral organ identity, and responding to environmental stimuli has garnered significant scientific interest in recent years [1,10,11,12]. This expanding research domain has dramatically shifted our understanding of lncRNAs, transcending their once presumed role as mere transcriptional byproducts to pivotal regulatory actors. In *Arabidopsis*, a model organism paramount for plant genetics and molecular biology research, the significance of lncRNAs in the flowering regulation network is increasingly evident. Flowering, a crucial juncture in plant development, unfolds under the precise governance of a complex array of genetic and epigenetic players, with lncRNAs being central to this regulatory ballet. One of the most distinguished lncRNAs in this realm is *COLD ASSISTED INTRONIC NONCODING RNA* (*COOLAIR*), an antisense lncRNA emanating from the *FLOWERING LOCUS C* (*FLC*) region. *FLC*, a principal inhibitor of flowering, is intricately modulated by *COOLAIR*. During vernalization—a requisite cold exposure for flowering in certain species—*COOLAIR*’s expression surges, leading to the repression of *FLC* and thus facilitating floral transition [13,14]. Another pivotal lncRNA in this regulatory landscape is *COLD ASSISTED INTRONIC NONCODING RNA* (*COLDAIR*), also originating from the *FLC* locus but in an alternate orientation relative to *COOLAIR*. *COLDAIR* plays a vital role in vernalization by interacting with the Polycomb Repressive Complex 2 (PRC2), catalyzing the trimethylation of histone H3 at lysine 27 (H3K27me3) at the FLC locus. This epigenetic alteration results in the enduring repression of *FLC*, paving the way for flowering induction [15]. Moreover, the discovery of lncRNAs like *FLOWERING BHLH DOWNREGULATED LONG INTERGENIC NONCODING RNA* (*FLORE*) further highlights the complexity of this regulatory network. *FLORE*, implicated in the photoperiodic pathway of flowering, is posited to modulate a cohort of genes, including flowering promoter FLOWERING BHLH 4 (FBH4). While the exact mechanism of *FLORE*’s action remains a subject of active investigation, its contribution to the intricate control of flowering timing is indisputable [16].

This background forms the basis for our investigation into the lncRNAs associated with *BrFLC* genes in Chinese cabbage (*Brassica rapa* L. ssp. *pekinensis*), a close relative of *Arabidopsis* and an important agricultural crop. The evolutionary kinship between these species suggests a possible conservation of lncRNA-mediated regulatory mechanisms, albeit with species-specific adaptations.

Building upon previous studies [17], we predicted the existence of lncRNAs associated with *BrFLC1* and *BrFLC2*. This study advances this field by cloning and characterizing three novel lncRNAs in Chinese cabbage. Employing a combination of quantitative expression analysis, in situ hybridization in Chinese cabbage shoot apex and leaves, and heterologous expression in *Arabidopsis*, we dissected their functional roles. This multifaceted approach allowed us exploration of the spatial and temporal expression patterns of these lncRNAs and their impact on the expression of *BrFLC* genes. Moreover, by generating transgenic *Arabidopsis* lines overexpressing these lncRNAs, we were able to observe altered flowering times and dissect the molecular pathways influenced by these lncRNAs. This heterologous system provided a valuable platform for validating the functional significance of these lncRNAs in flowering regulation.

## 2. Results

### 2.1. Identification of lncFLC1, lncFLC2a and lncFLC2b in Chinese Cabbage

**Figure 1:**
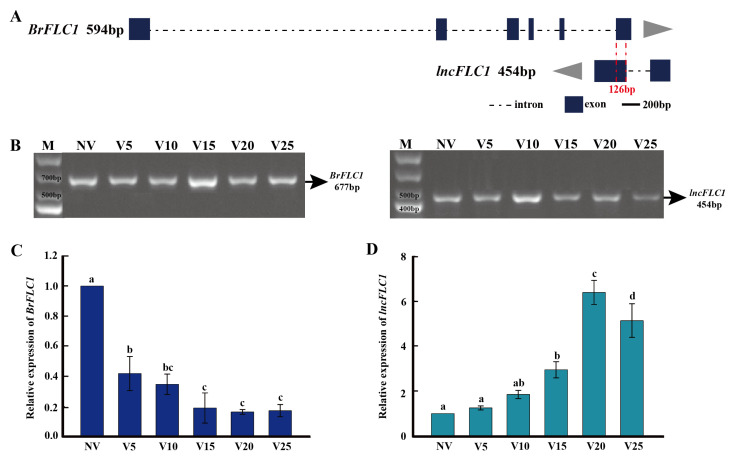
Identification of *lncFLC1*. (**A**) Depicts the genomic location schematic of *BrFLC1* and *lncFLC1*. The depicted gene structure includes sizes in base pairs (bp) representing the length of the cDNA. Blue blocks represent exons, dashed lines indicate introns, and the red 126bp denotes the complementary region between *BrFLC1* and *lncFLC1.* (**B**) Illustrates the PCR product of *BrFLC1* and *lncFLC1*. (**C**) Presents RT-qPCR analysis of *BrFLC1* expression across various vernalization stages. (**D**) Presents RT-qPCR analysis of *lncFLC1* expression across various vernalization stages. NV represents “non-vernalized”, while V5, V10, V15, V20, and V25 indicate 5, 10, 15, 20, and 25 days of vernalization, respectively. Error bars represent the SE (*n* = 3). Values with the same letter are not significantly different at the *p* < 0.05 level.

**Figure 2:**
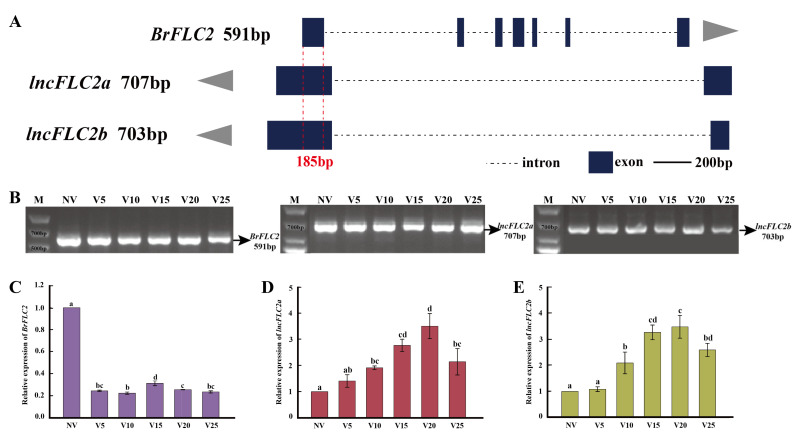
Identification of *lncFLC2a* and *lncFLC2b*. (**A**) Depicts the genomic location schematic of *BrFLC2, lncFLC2a* and *lncFLC2b*. The depicted gene structure includes sizes in base pairs (bp) representing the length of the cDNA. Blue blocks represent exons, dashed lines indicate introns, and the red 185bp denotes the complementary region between *BrFLC2* and *lncFLC2.* (**B**) Illustrates the PCR product of *BrFLC2, lncFLC2a* and *lncFLC2b*. (**C**) Presents RT-qPCR analysis of *BrFLC2* expression across various vernalization stages. (**D**) Presents RT-qPCR analysis of *lncFLC2a* expression across various vernalization stages. (**E**) Presents RT-qPCR analysis of *lncFLC2b* expression across various vernalization stages. NV represents “non-vernalized”, while V5, V10, V15, V20, and V25 indicate 5, 10, 15, 20, and 25 days of vernalization, respectively. Error bars represent the SE (*n* = 3). Values with the same letter are not significantly different at the *p* < 0.05 level.

In our initial transcriptome analysis of the Chinese cabbage “JHX” during vernalization, we predicted antisense lncRNAs related to *BrFLC1* and *BrFLC2* [17]. Using primers derived from sequences obtained via transcriptome sequencing, we conducted cloning analyses of *BrFLC1*, *BrFLC2*, and their corresponding antisense lncRNAs. The lncRNA of *BrFLC1* is composed of two exons and one intron, with a total DNA length of 659 bp and a cDNA length of 454 bp. It features a 126 bp base complementary region with the last exon of *BrFLC1*, and it is named *lncFLC1* (Figure 1A,B). From the five antisense lncRNAs related to *BrFLC2* predicted in our transcriptome analysis, we successfully cloned two, named *lncFLC2a* and *lncFLC2b*. Both lncRNAs are composed of two exons and one intron. *LncFLC2a* has a DNA length of 3815 bp and a cDNA length of 707 bp, sharing a 185 bp base complementary region with the first exon of *BrFLC2*. Similarly, *lncFLC2b* has a DNA length of 3888 bp and a cDNA length of 703 bp, also featuring a 185 bp complementary base region with the first exon of *BrFLC2* (Figure 2A,B).

### 2.2. Vernalization-Induced Upregulation of lncFLC1, lncFLC2a, and lncFLC2b

LncRNAs are characterized by their time- and tissue-specific transcriptional responses [18,19,20]. Using real-time quantitative polymerase chain reaction (RT-qPCR), we meticulously investigated the spatiotemporal expression dynamics of *BrFLC1*, *BrFLC2*, and their corresponding lncRNAs. Notably, *BrFLC1* expression exhibited a consistent downward trend from the non-vernalization (NV) phase extending to 25 days of vernalization (V25) (Figure 1C). In stark contrast, *lncFLC1*’s expression pattern was inverse, showing a progressive increase up to the V20 mark within the vernalization period, which was then followed by a minor reduction at V25 (Figure 1D).

*BrFLC2* exhibits a pronounced vernalization response, with a marked reduction in expression at the V5 stage relative to non-vernalized (NV) conditions, followed by consistently low expression levels throughout vernalization (Figure 2C). *LncFLC2a* and *LncFLC2b*, akin to *lncFLC1*, exhibit a gradual increase in expression from NV to V20, then a slight decline from V20 to V25 (Figure 2D,E). Interestingly, these three cloned lncRNAs demonstrate expression patterns that are inversely related to their target genes during vernalization (Figure 1C,D and Figure 2C–E). In *Arabidopsis*, *AtFLC*’s lncRNA *COOLAIR* displays a base-complementary pattern and a reverse change in expression during vernalization, thereby lowering *AtFLC* expression and facilitating rapid flowering. It is hypothesized that *lncFLC1*, *lncFLC2a*, and *lncFLC2b* may function similarly to *COOLAIR*, targeting *BrFLC1* and *BrFLC2* to reduce their expression levels.

### 2.3. Expression Profiles of lncFLC1 and lncFLC2 

**Figure 3:**
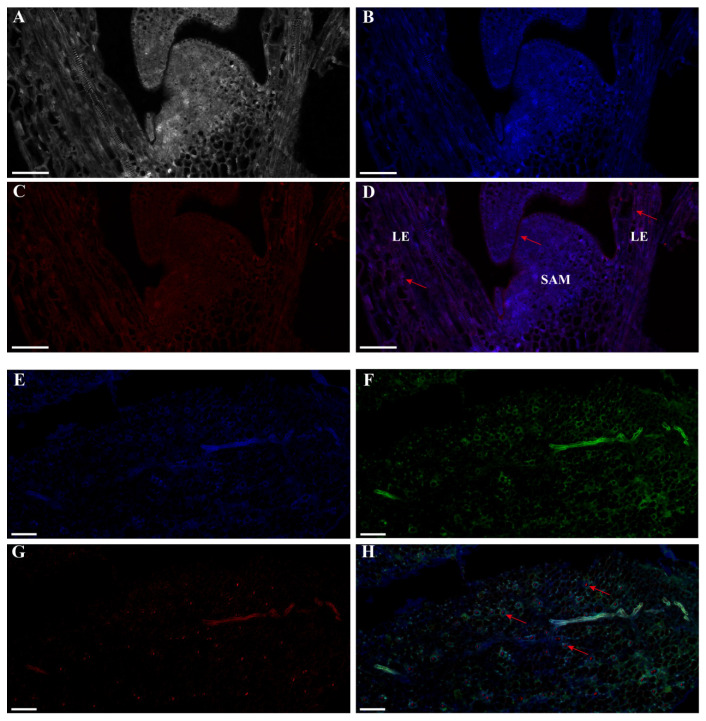
Depicting localization transcription patterns of *lncFLC1* through fluorescent in situ hybridization (FISH). (**A**–**D**) Single-label FISH localization for *lncFLC1* in the shoot apex of Chinese cabbage. (**E**–**H**) Dual-label FISH localization analysis for both *BrFLC1* with *lncFLC1* in the leaves of Chinese cabbage. Blue signal: nuclear localization; green signal: *BrFLC1* localization; red signal: *lncFLC1* localization. Arrows highlight specific regions demonstrating colocalization. Long scale bar = 50 µm; short scale bar = 20 µm; SAM: shoot apical meristem; LE: leaf or leaf primordia.

**Figure 4:**
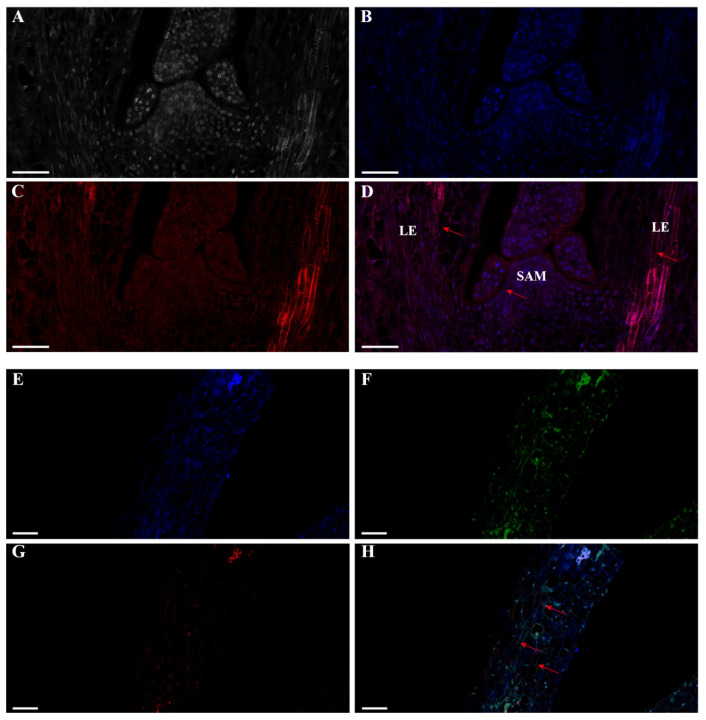
Depicting localization transcription patterns of *lncFLC2* through fluorescent in situ hybridization (FISH). (**A**–**D**) Single-label FISH localization for *lncFLC2* in the shoot apex of Chinese cabbage. (**E**–**H**) Dual-label FISH localization analysis for both *BrFLC2* with *lncFLC2* in the leaves of Chinese cabbage. Blue signal: nuclear localization; green signal: *BrFLC2* localization; red signal: *lncFLC2* localization. Arrows highlight specific regions demonstrating colocalization. Long scale bar = 50 µm; short scale bar = 20 µm; SAM: shoot apical meristem; LE: leaf or leaf primordia.

Comprehending the transcriptional dynamics of lncRNAs is vital for elucidating their regulatory functions in plants [21,22,23]. We used the V25 sample of Chinese cabbage to conduct single-label FISH localization for *lncFLC1* (Figure 3A–D) and *lncFLC2* (Figure 4A–D). Additionally, we performed dual-label FISH localization analysis for both *BrFLC1* with *lncFLC1* (Figure 3E–H) and *BrFLC2* with *lncFLC2* (Figure 4E–H). In these analyses, nuclear localization was indicated by a blue signal, *BrFLC* localization by a green signal, and *lncFLC* localization by a red signal. 

*LncFLC1* and *lncFLC2* showed extensive distribution in the shoot apical meristem (SAM) of Chinese cabbage (Figure 3A–D and Figure 4A–D). Dual-label FISH analyses indicated non-overlapping localization in leaf tissues for *BrFLC1* with *lncFLC1* and *BrFLC2* with *lncFLC2*. Notably, *BrFLC1* surrounded the *lncFLC1* region (Figure 3H), and *BrFLC2* was in close vicinity to *lncFLC2* (Figure 4H), suggesting that these lncRNAs are closely associated with their target gene *BrFLC*, thereby influencing its regulatory function.

### 2.4. Expression Patterns of lncFLC1, lncFLC2a and lncFLC2b in Arabidopsis Transgenic Lines

**Figure 5:**
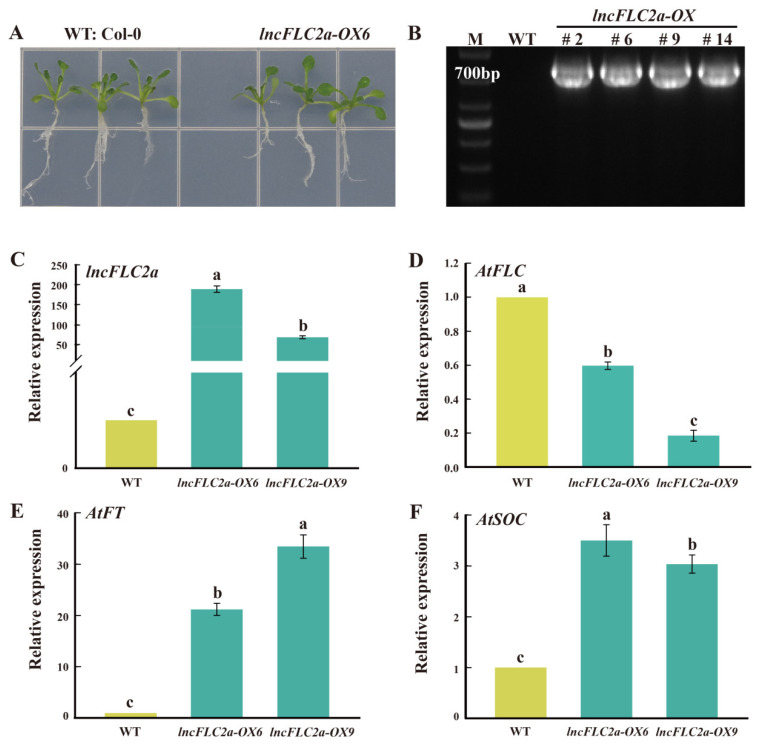
Identification of *lncFLC2a* overexpression lines and analysis of its expression pattern. (**A**) Phenotypes of *lncFLC2a* and WT plants grown in medium for 10 days. (**B**) The *lncFLC2a* coding sequence from the *lncFLC2a*-OX lines were cloned by PCR. (**C**–**F**) Relative expression of *lncFLC2a* and the flowering-related genes *AtFLC*, *AtFT* and *AtSOC* in *lncFLC2a*-OX and WT plants. Error bars represent the SE (*n* = 3). Values with the same letter are not significantly different at the *p* < 0.05 level, WT represents Columbia wild type; OX represents overexpression lines.

**Figure 6:**
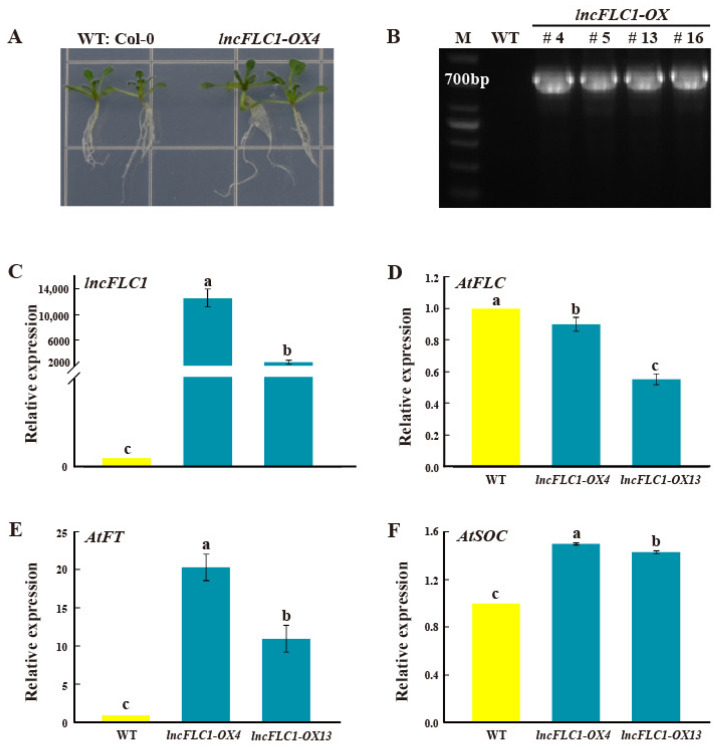
Identification of *lncFLC1* overexpression lines and analysis of its expression pattern. (**A**) Phenotypes of *lncFLC1* and WT plants grown in medium for 10 days. (**B**) The *lncFLC1* coding sequences from the *lncFLC1*-OX lines were cloned by PCR. (**C**–**F**) Relative expression of *lncFLC1* and the flowering-related genes *AtFLC*, *AtFT* and *AtSOC* in *lncFLC1*-OX and WT plants. Error bars represent the SE (*n* = 3). Values with the same letter are not significantly different at the *p* < 0.05 level, WT represents Columbia wild type; OX represents overexpression lines.

**Figure 7:**
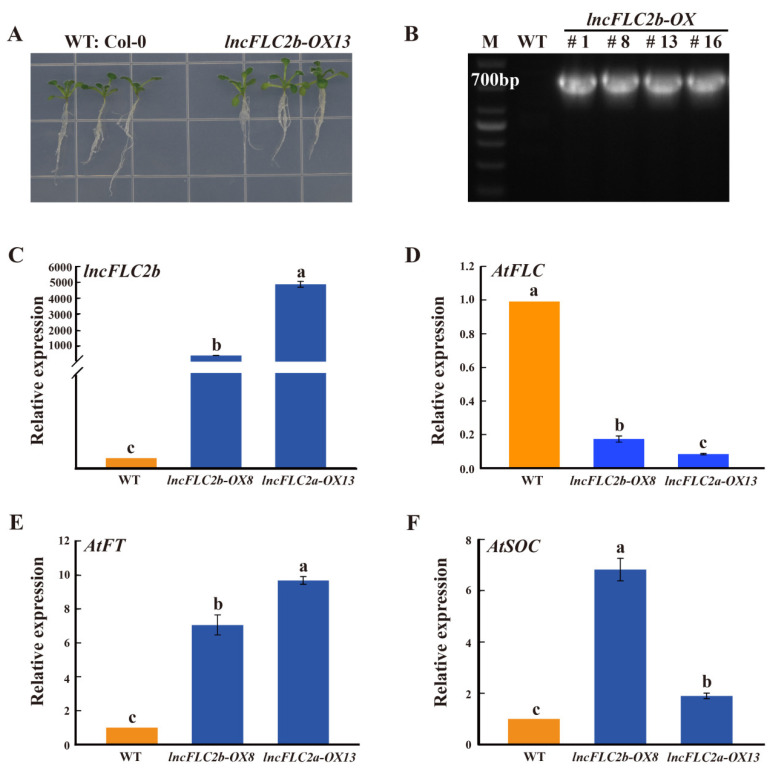
Identification of *lncFLC2b* overexpression lines and analysis of its expression pattern. (**A**) Phenotypes of *lncFLC2b* and WT plants grown in medium for 10 days. (**B**) The *lncFLC2b* coding sequence from the *lncFLC2b*-OX lines were cloned by PCR. (**C**–**F**) Relative expression of *lncFLC2b* and the flowering-related genes *AtFLC*, *AtFT* and *AtSOC* in *lncFLC2b*-OX and WT plants. Error bars represent the SE (*n* = 3). Values with the same letter are not significantly different at the *p* < 0.05 level, WT represents Columbia wild type; OX represents overexpression lines.

Due to the absence of a stable gene transformation system in Chinese cabbage, we undertook the transient overexpression of *lncFLC1*, *lncFLC2a*, and *lncFLC2b* in *Arabidopsis* seedlings. This approach was employed to ascertain the potential role of these three lncRNAs in facilitating the flowering process. We expressed *lncFLC1*, *lncFLC2a*, and *lncFLC2b* in *Arabidopsis* under the control of the CaMV35S promoter. Through kanamycin resistance selection and PCR analysis, stable homozygous T3 transgenic lines were progressively established. In the comparative cultivation of these T3 lines with the Columbia wild type (WT) on 1/2 MS medium, it was noted that *lncFLC2a* exhibited no discernible phenotypic variances from WT after a growth period of 10 days (Figure 5A). In contrast, *lncFLC1* and *lncFLC2b* demonstrated a marginally larger size compared to WT (Figure 6A and Figure 7A). Genomic PCR analysis substantiated the integration of *lncFLC1*, *lncFLC2a*, and *lncFLC2b* into the *Arabidopsis* genome (Figure 5B, Figure 6B and Figure 7B). In the T3 lines, overexpression markedly elevated the expression levels of *lncFLC1*, *lncFLC2a*, and *lncFLC2b* (Figure 5C, Figure 6C and Figure 7C). To assess the impact of these lncRNAs on the expression of pivotal *Arabidopsis* flowering genes, we examined three critical genes governing flowering in *Arabidopsis* (*AtFLC*, *AtFT*, and *AtSOC*). *AtFLC*, known as a flowering suppressor [24,25], exhibited decreased expression across all three lncRNA overexpression lines (Figure 5D, Figure 6D and Figure 7D). Conversely, *AtFT* and *AtSOC*, known to facilitate flowering genes [26,27,28], demonstrated increased expression in these overexpression lines (Figure 5E,F, Figure 6E,F and Figure 7E,F). This pattern suggests that the overexpression of *lncFLC1*, *lncFLC2a*, and *lncFLC2b* significantly influences the expression of various *Arabidopsis* flowering genes.

### 2.5. Promotion of Early Flowering in Arabidopsis by Overexpression of lncFLC1, lncFLC2a and lncFLC2b

**Figure 8:**
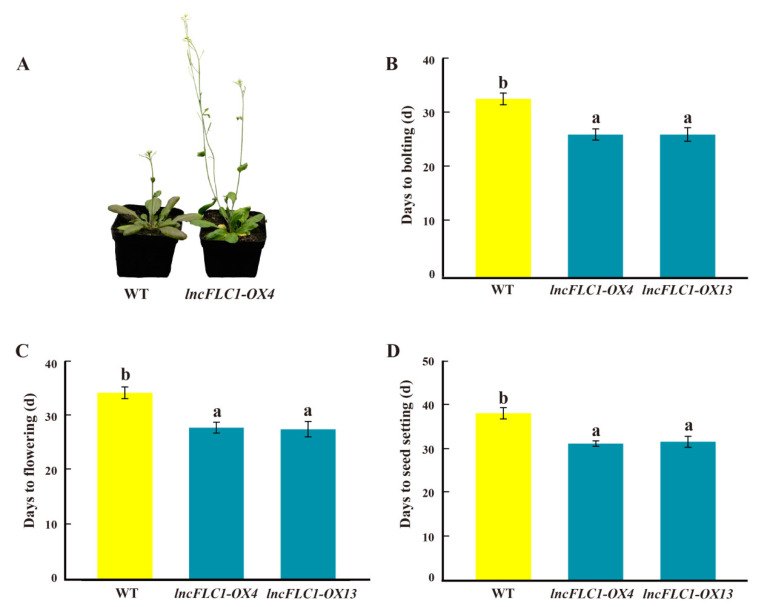
Phenotypic investigation of *lncFLC1* overexpression lines. (**A**) Phenotypes of *lncFLC1*-OX and WT plants observed 35 days post planting. (**B**–**D**) Statistics on the days to bolting, flowering and seed setting of *lncFLC1*-OX and WT plants. Error bars represent the SE (*n* = 10). Values with the same letter are not significantly different at the *p* < 0.05 level, WT represents Columbia wild type; OX represents overexpression lines.

**Figure 9:**
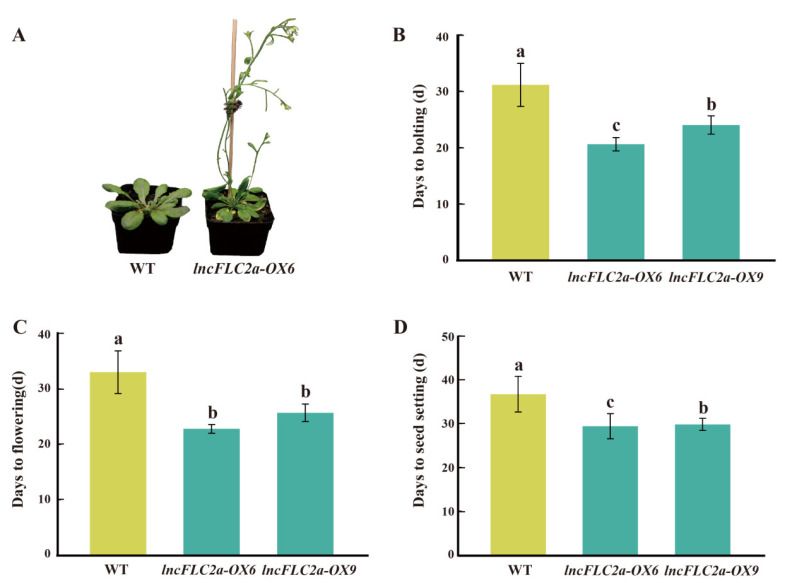
Phenotypic investigation of *lncFLC2a* overexpression lines. (**A**) Phenotypes of *lncFLC2a*-OX and WT plants observed 30 days post planting. (**B**–**D**) Statistics on the days to bolting, flowering and seed setting of *lncFLC2a*-OX and WT plants. Error bars represent the SE (*n* = 10). Values with the same letter are not significantly different at the *p* < 0.05 level, WT represents Columbia wild type; OX represents overexpression lines.

**Figure 10:**
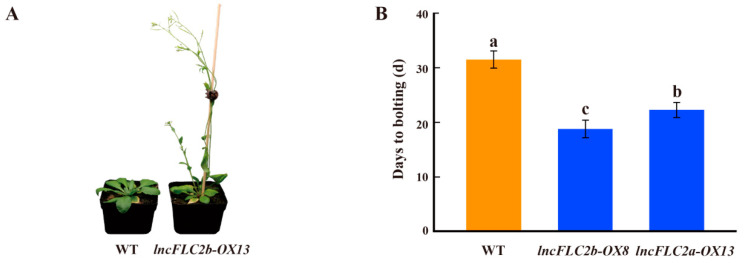

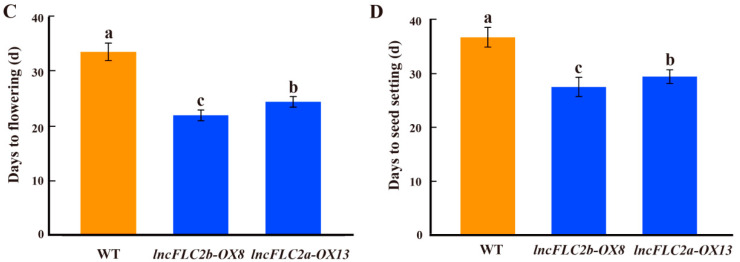
Phenotypic investigation of *lncFLC2b* overexpression lines. (**A**) Phenotypes of *lncFLC2b*-OX and WT plants observed 30 days post planting. (**B**–**D**) Statistics on the days to bolting, flowering and seed setting of *lncFLC2b*-OX and WT plants. Error bars represent the SE (*n* = 10). Values with the same letter are not significantly different at the *p* < 0.05 level, WT represents Columbia wild type; OX represents overexpression lines.

In this study, the overexpression lines of *lncFLC1*, *lncFLC2a*, and *lncFLC2b* in *Arabidopsis* demonstrated a remarkable acceleration in developmental phases when compared to the WT, as evidenced by earlier bolting, flowering, and seed setting (Figure 8, Figure 9 and Figure 10). This acceleration is indicative of a profound influence of these lncRNAs on the plant’s development. Significantly, while the overexpression of *lncFLC1* induced earlier bolting and flowering, its impact was somewhat subtler than that observed for *lncFLC2a* and *lncFLC2b*. Specifically, *lncFLC1* overexpression lines exhibited bolting approximately six days earlier than the WT (Figure 8B), suggesting a nuanced yet effective role in advancing the flowering process. In contrast, the overexpression lines of *lncFLC2a* and *lncFLC2b* showed a more pronounced advancement in developmental stages. These lines not only initiated bolting and flowering earlier but also progressed to seed setting, a stage not reached by the WT plants, which remained in the vegetative phase. This advancement was approximately ten days ahead of the WT (Figure 9B and Figure 10B), highlighting a potent acceleration effect of these lncRNAs on floral development. The distinct developmental trajectories observed in the overexpression lines of *lncFLC1*, *lncFLC2a*, and *lncFLC2b* provide compelling evidence of the significant and diverse roles these lncRNAs play in regulating flowering in *Arabidopsis*. The variation in the extent of acceleration between the overexpression lines underscores the possibility of intricate, lncRNA-specific mechanisms influencing the timing and progression of flowering. Overall, the accelerated flowering phenotype across all three lncRNA overexpression lines underscores their potential as key regulatory elements in the flowering process of *Arabidopsis*. These findings not only augment our understanding of the genetic and molecular underpinnings of flowering, but also open up new avenues for exploring the functional diversity and potential applications of lncRNAs in plant developmental biology. Our data indicate that *lncFLC1*, *lncFLC2a*, and *lncFLC2b* may be crucial components of a complex regulatory network, modulating key aspects of the flowering process in Chinese cabbage. These findings align with our research aim to explore how *BrFLC*-associated lncRNAs regulate the flowering response in Chinese cabbage. 

## 3. Discussion

This study aims to explore the regulatory dynamics of *BrFLC*-associated lncRNAs in modulating the flowering response of Chinese cabbage, with a particular focus on *lncFLC1*, *lncFLC2a*, and *lncFLC2b* during the vernalization process. While previous studies have identified lncRNAs expressed in Chinese cabbage under vernalization conditions [29,30,31,32], our research provides new insights by directly exploring the interactions between these lncRNAs and *BrFLC* genes, contributing to our understanding of the unique mechanisms of lncRNAs in regulating flowering in Chinese cabbage. This research illuminates the possible regulatory mechanisms of these lncRNAs, drawing parallels to the well-established *COOLAIR* pathway in *Arabidopsis*, as referenced in earlier studies [14,15]. Our comprehensive analysis reveals a remarkable trend: during vernalization, there is a pronounced upsurge in the expression levels of *lncFLC1*, *lncFLC2a*, and *lncFLC2b*. This increase is intricately linked with a simultaneous downregulation of *BrFLC1* and *BrFLC2*, which are pivotal floral repressors in Chinese cabbage (Figure 1 and Figure 2) [33,34]. This inverse expression pattern is not merely coincidental; it strongly hints at a sophisticated regulatory interplay. We hypothesize that this relationship could operate through a mechanism akin to that of *COOLAIR* [14,15]. In the *COOLAIR* model, antisense-mediated regulation of *FLC* is a key player in determining the timing of flowering. 

In the intricate world of plant biology, *Arabidopsis* has served as a model organism for understanding the genetic control of flowering time [35,36]. *COOLAIR*, an antisense transcript to *FLC*, plays a pivotal role in modulating *FLC* expression and thereby influencing the timing of flowering. Intriguingly, during the cold-induced vernalization process, an upregulation of *COOLAIR* is observed, leading to a consequential downregulation of FLC, a key floral repressor. This modulation facilitates floral transition, a critical phase in plant development [14,15]. While our findings reveal certain parallels, they also underscore fundamental differences in the flowering regulation mechanisms between Chinese cabbage and *Arabidopsis*, suggesting species-specific adaptations in lncRNA functions [37,38]. Utilizing in situ hybridization experiments, we observed a spatial expression pattern for *lncFLC1* and *lncFLC2*, along with *BrFLC1* and *BrFLC2*, that mirrors the pattern seen in *Arabidopsis*. Particularly notable was the expression in the shoot apical meristem and leaves, the primary sites for floral induction (Figure 3 and Figure 4). These insights suggest a potential functional homology between these lncRNAs in Chinese cabbage and *COOLAIR* in *Arabidopsis*. It is conceivable that, similar to *COOLAIR*, these lncRNAs in Chinese cabbage might serve as critical modulators of flowering time regulation. This could occur through a mechanism involving antisense transcriptional interference or chromatin remodeling, a hypothesis that aligns with the current understanding of gene regulation mechanisms in plants [39].

Further supporting this hypothesis, the heterologous overexpression of *lncFLC1*, *lncFLC2a*, and *lncFLC2b* in *Arabidopsis* led to an accelerated flowering phenotype, including earlier bolting, flowering, and seed-setting (Figure 8, Figure 9 and Figure 10). This phenotypic similarity to *Arabidopsis* lines overexpressing *COOLAIR* reinforces the notion that the functional roles of these lncRNAs in flowering time regulation are conserved across species. The detailed phenotypic analysis and gene expression studies in *Arabidopsis* provided insights into how these lncRNAs might interact with the *Arabidopsis* flowering regulatory network, possibly mimicking the functional aspects of *COOLAIR*. Simultaneously, the heterologous overexpression of *lncFLC1*, *lncFLC2a*, and *lncFLC2b* in *Arabidopsis* notably reduced the expression of flowering repressor gene *AtFLC*. Concurrently, there was an elevation in the expression levels of flowering-promoting genes *AtFT* and *AtSOC1* (Figure 5, Figure 6 and Figure 7). This indicates that these lncRNAs may actively participate in the modulation of key genetic pathways, influencing the balance between flowering repression and promotion, and thereby playing a critical role in the regulation of the flowering process in *Arabidopsis*. The accelerated flowering observed in *Arabidopsis* upon overexpression of these lncRNAs raises intriguing questions about their potential role in the modulation of flowering pathways, both in their native Chinese cabbage and in *Arabidopsis*. Their influence on the expression levels of key flowering genes, similar to the role of *COOLAIR* in repressing *FLC*, suggests a shared evolutionary strategy among Brassicaceae species for flowering time regulation through lncRNAs.

In conclusion, our study not only expands the understanding of lncRNA-mediated flowering regulation in Chinese cabbage, but also provides compelling evidence for the functional conservation of these regulatory mechanisms across species. Importantly, our findings support a new perspective that, despite the absence of lncRNAs associated with vernalization response in *Arabidopsis* in Chinese cabbage [32,40,41], it might possess its independent mechanism for flowering control, offering a new direction for future research. This research opens new avenues for exploring the role of lncRNAs in plant development, *lncFLC1*, *lncFLC2a*, and *lncFLC2b* reflecting a mechanism similar to the *COOLAIR* in *Arabidopsis*, thereby uncovering a potential universal flowering regulation strategy across related species. Practically, our findings hold significant promise for agricultural applications. Understanding flowering control mechanisms at the molecular level in Chinese cabbage has direct implications for crop management and breeding strategies. The identification of *lncFLC1*, *lncFLC2a*, and *lncFLC2b* as key regulators resembling the *COOLAIR* mechanism in *Arabidopsis* suggests potential targets for crop improvement. Manipulating these lncRNAs may lead to the development of novel approaches for precisely controlling flowering in Chinese cabbage varieties, thereby optimizing crop production. In summary, this research not only advances our theoretical knowledge of lncRNA-mediated flowering regulation, but also offers practical avenues for enhancing agricultural practices. It underscores the importance of exploring the roles of lncRNAs in plant development, paving the way for innovative strategies in crop management and breeding.

## 4. Materials and Methods

### 4.1. Plant Material and Growth Conditions

Ju Hongxin (JHX), a Chinese cabbage cultivar, was obtained from the Institute of Vegetables and Flowers, Chinese Academy of Agricultural Sciences. Uniformly developed seeds of JHX were initially rinsed with sterile water and then arrayed on petri dishes lined with dual layers of filter paper. For germination enhancement, the seeds were incubated in a climatic chamber maintained at a steady 25 °C, with a photoperiod of 16 h light per day, for a duration of two days. After the radicles emerged through the seed coat, the seeds were transferred to a vernalization chamber set at 4 °C. Here, they were subjected to a 22/2 h light/dark cycle with a light intensity of 150 µmol m^−2^ s^−1^ for a period of 25 days. Sampling was meticulously conducted at designated vernalization stages: non-vernalized (NV) and after 5 (V5), 10 (V10), 15 (V15), 20 (V20), and 25 (V25) days of vernalization. At each stage, we collected specific tissues from the shoot apex. All samples were promptly snap-frozen in liquid nitrogen and subsequently stored at −80 °C for preservation. This approach ensured a comprehensive analysis of lncRNA expression patterns, thereby providing a more nuanced understanding of their roles during vernalization.

Seeds of *Arabidopsis* WT and transgenic lines underwent surface sterilization sequentially in 75% (*v*/*v*) ethanol for 30 s, followed by 2% (*v*/*v*) sodium hypochlorite for 10 min. After three rinses with sterile distilled water, the seeds were germinated on 1/2 Murashige and Skoog (MS) medium (comprising 1% sucrose and 0.7% agar, pH 5.8). They were cultivated in a greenhouse under a 10/14 h light/dark cycle at a light intensity of 150 µmol m^−2^ s^−1^ for a period of 10 days. Post germination, seedlings were transplanted into nutrient-rich soil for further growth, facilitating subsequent seed harvesting, sampling, and phenotypic analysis.

### 4.2. RNA Fluorescence In Situ Hybridization (FISH) Technique

The cDNA from the “JHX” V25 stage was utilized as a template for cloning the full-length cDNAs of *BrFLC1*, *BrFLC2*, *lncFLC1*, *lncFLC2a*, and *lncFLC2b*. Specifically designed labeled nucleic acid probes were used for detecting the cellular localization of *lncFLC1*, *lncFLC2a*, and *lncFLC2b*. In addition, specifically designed probes were used to detect the colocalization of *BrFLC1* with *lncFLC1* and *BrFLC2* with *lncFLC2*, targeting complementary sequences in the shoot apex and leaves of Chinese cabbage. The shoot apex tissue and leaf from “JHX” V25 samples was first sectioned and then submerged in RNase-free formaldehyde fixative to ensure preservation. Dehydration of the shoot apex and leaf tissues was achieved using the JJ-12J system (WHJJ, Wuhan, China), a process specifically intended for tissue dehydration. This was followed by embedding and slicing using the JB-P5 system (WHJJ, Wuhan, China) and a Leica RM2016 rotary slicer (Leica, Shanghai, China). Subsequently, the tissue slices were washed with PBS containing 0.5% TritonX-100 to remove any wax before undergoing hybridization with the probes in a hybridization solution. Detection of the labeled probes was carried out under an inverted fluorescence microscope (NIKON, Tokyo, Japan). The sections were then scanned into slides using an automatic slide scanner (3DHISTECH, Budapest, Hungary). The sequences of the primers and probes used are provided in Supplementary Table S1.

### 4.3. Construction of Plasmids and Generation of Transgenic Arabidopsis Lines

The cDNA sequences of *lncFLC1*, *lncFLC2a*, and *lncFLC2b* (Supplementary Table S2) were inserted into the pBI121 vector, positioned under the cauliflower mosaic virus (CaMV) 35S promoter, thus constructing plasmids 35S::*lncFLC1*, 35S::*lncFLC2a*, and 35S::*lncFLC2b*. After transplantation into potting soil, *Arabidopsis* WT plants were cultivated in a greenhouse environment. The growth conditions were maintained at a 10/14 h light/dark cycle with a light intensity of 150 µmol m^−2^ s^−1^, continuing until the onset of bolting and flowering. Following this, the aforementioned plasmids were introduced into *Arabidopsis* plants by employing the floral dip technique [42]. Screening of T0 transgenic *Arabidopsis* for the 35S::*lncFLC1*, 35S::*lncFLC2a*, and 35S::*lncFLC2b* constructs was performed on 1/2 MS medium infused with 25 µg mL^−1^ kanamycin. The identification of T1 transformants was based on the analysis of seeds from T0 *Arabidopsis*, adhering to the (3:1) kanamycin resistance segregation ratio. This rigorous screening process culminated in the selection of independent T3 homozygous lines, which were then utilized in further experimental investigations. The integrity and authenticity of the transgenic plants were corroborated through PCR analysis, utilizing specific primers targeted for *lncFLC1*, *lncFLC2a*, and *lncFLC2b*. The sequences of the primers used are provided in Supplementary Table S1.

### 4.4. Phenotypic Investigation of Transgenic Arabidopsis Plants

After a 10-day cultivation period on 1/2 MS medium, OX lines and WT *Arabidopsis* seeds were transplanted to potting soil and subsequently grown in a controlled greenhouse environment. The growth conditions were meticulously maintained under a 10/14 h light/dark cycle with a light intensity of 150 µmol m^−2^ s^−1^. The initiation of this growth phase in potting soil was designated as Day 0. Key developmental stages, including the bolting stage identified when the shoot reached a height of 2 cm, the flowering stage marked by the full bloom of the first flower, and the seed-setting stage denoted upon the complete development of the first pod were closely monitored and recorded. This experiment was methodically replicated across ten biological repeats for each treatment to ensure data robustness and reproducibility.

### 4.5. Gene Expression Analysis 

Total RNA from plant specimens was meticulously extracted employing the TransZol Up Plus RNA Kit (TransGen, Beijing, China) and subsequently treated with RNase-free DNase I (TransGen, Beijing, China). Approximately 2 µg of total RNA was reverse-transcribed into cDNA using the TransScript^®^ IV Reverse Transcriptase, followed by a 1:15 dilution of the cDNA for deployment in both polymerase chain reaction (PCR) and quantitative real-time polymerase chain reaction (qRT-PCR) assays. The expression levels of *BrFLC1*, *BrFLC2*, *lncFLC1*, *lncFLC2a* and *lncFLC2b* in Chinese cabbage were based on shoot apex tissues at six stages: non-vernalized (NV) and after 5 (V5), 10 (V10), 15 (V15), 20 (V20), and 25 (V25) days of vernalization. In addition, the expression levels of *lncFLC1*, *lncFLC2a* and *lncFLC2b* in *Arabidopsis* overexpression lines were assessed based on samples collected when the *Arabidopsis* shoot reached a height of 2 cm. Primers, predicated on the sequences of *BrFLC1*, *BrFLC2*, *lncFLC1*, *lncFLC2a*, and *lncFLC2b* were synthesized and employed in PCR. The amplified fragments, retrieved from agarose gel, underwent sequencing for comparative analyses. In qRT-PCR, primers targeting approximately 150 bp DNA segments of the aforementioned *lncFLC* sequences were utilized, with each reaction comprising five replicates. The CFX-96 Real-time System (BIORAD, Hercules, CA, USA) was harnessed for qRT-PCR procedures. Employing the internal Actin of Chinese cabbage and *Arabidopsis* as internal controls, gene expression data were rigorously analyzed using the 2^−ΔCt^ method [43]. The sequences of the primers used are provided in Supplementary Table S1.

### 4.6. Statistical Analysis

Statistical evaluations, specifically one-way analysis of variance (ANOVA) complemented by Duncan’s multiple range post hoc test (significance threshold set at *p* < 0.05), were conducted using SPSS v19.0 (SPSS, Chicago, IL, USA). Lastly, the results were elegantly visualized employing Sigmaplot v10.0 (Systat Software Inc., San Jose, CA, USA).

## Data Availability

Data are contained within the article or Supplementary Materials.

## Supplementary Materials

The following supporting information can be downloaded at: https://www.mdpi.com/article/10.3390/ijms25031924/s1.
